# Mycobiota and mycotoxin-producing fungi in southern California: their colonisation and *in vitro* interactions

**DOI:** 10.1080/21501203.2022.2104950

**Published:** 2022-07-27

**Authors:** Paris Chakravarty

**Affiliations:** Department of Microbiology, Pasteur Laboratory, Glendora, CA, USA

**Keywords:** Indoor and outdoor air-borne fungi, *in vitro* interactions, mycotoxins, fungal colonisation

## Abstract

Fungal contamination in water-damaged houses has become a major concern because of their potential health effects. During our survey in 100 water-damaged houses in southern California, we have reported 42 outdoor and 14 indoor fungal species throughout the year. Six commonly occurring indoor fungal species are *Alternaria alternata, Aspergillus niger, Chaetomium globosum, Cladosporium herbarum, Penicillium chrysogenum* and *Stachybotrys chartarum*. In the damp building materials, *S. chartarum* was found to be associated with *A. niger, C. globosum* and *P. chrysogenum* but not with *A. alternata* and *C. herbarum. Stachybotrys chartarum* showed strong antagonistic effect against *A. alternata* and *C. herbarum* and significantly inhibited *in vitro* growth of *A. alternata* and *C. herbarum* but had no effect on *A. niger, C. globosum* and *P. chrysogenum*. Two trichothecenes, produced by *Stachybotrys* sp., trichodermin and trichodermol, significantly inhibited spore germination and *in vitro* growth of *A. alternata* and *C. herbarum* but had no effect on *A. niger, C. globosum, P. chrysogenum* and *S. chartarum*. In the damp building materials (drywall, ceiling tile, and oak wood), *S. chartarum* significantly inhibited the growth of *A. alternata* and *C. herbarum* and had no effect on *A. niger, C. globosum and P. chrysogenum* in these substrata.

## 1. Introduction

Although fungi are ubiquitous, not all the fungi grow in the same environment. The population of fungal species differ significantly in outdoor and indoor environments (Andersen et al. 2011, 2017, 2021). Certain fungi such as species of *Cladosporium* and *Penicillium* can grow both in wet and semi-dry materials, whereas species of *Chaetomium* and *Stachybotrys* need wet building materials to grow and multiply. Fungal exposures in damp or water-damaged houses have become a major concern because of their potential health effects and there is a clear relationship between contaminated indoor environments and illness. Indoor air quality became an important issue since 1960s when researchers found that indoor pollutant levels in water-damaged houses can reach or exceed those of outdoor levels (Anderson et al. 1997; Shelton et al. 2002). It is now well-known fact that indoor air quality is an essential part of our health since we spend 90% of our time indoors inhaling approximately 15 m^3^ of ambient air every day (Sundell 2004). The occupants of wet, mouldy buildings have increase in subjective complaints and children in damp homes show higher respiratory and other illness (Montgomery et al. 1989; Anderson et al. 1997; Hyvarinen et al. 2002). It is reported that fungi colonising in one area of the home can spread and contaminate the entire home (Hegarty et al. 2019). The list of symptoms generally consists of upper respiratory complaints, including headache, eye irritation, epistaxis, nasal and sinus congestion, cough, and gastrointestinal complaints (Platt et al. 1989). The exposure to fungal spores enhances the histamine release triggered by both allergic and non-immunologic mechanisms in the cultured leukocytes (Mahmoiudi and Gershwin 2000). Besides water damage, high temperature and relative humidity can also contribute to the higher occurrence of indoor fungi in the house (Christensen et al. 1995; Davies et al. 1995; Burge 2004).

We have studied 100 water-damaged houses in Southern California and compared the occurrence of fungal flora both inside and outside of houses for a period of one year. In this study, it was found that the fungal population varied significantly in outdoor and indoor damp environments and certain fungi occur in both the environments. Six most commonly occurring fungal species in the indoor air and damp building materials were *Alternaria alternata* (Fr.) Keissl., *Aspergillus niger* van Tieghem, *Chaetomium globosum* Kunze ex Steud., *Cladosporium herbarum* (Pers.) Link ex Gray, *Penicillium chrysogenum* Thom and *Stachybotrys chartarum* (Ehrenb. ex Link) Hughes (Chakravarty and Kovar 2013). It was found that certain fungi occur alone or in combination with other fungi. Interactions between the different fungi in a water-damaged house are unavoidable because spores of a single fungal species alone may contain various metabolites, and water-damaged site is always a habitat of more than one fungal species (Anderson et al. 1997; Nielsen et al. 2001). Many mycotoxins are thought to be involved in chemical signalling between organisms or species and the production of some of the mycotoxins may be stimulated or inhibited when microorganisms interact with each other (Nielsen et al. 2001). It was found that *S. chartarum* was growing alone or in combination with *A. niger, C. globosum* and *P. chrysogenum* in the damp or water-damaged building materials but not with *A. alternata* and *C. herbarum*. The frequent occurrence of *S. chartarum* with *A. niger, C. globosum* and *P. chrysogenum* in water-damaged building materials means that they often share their habitat in common damp or water-damaged building materials without any effect on the mycotoxin produced by *S. chartarum. Stachybotrys chartarum* produces secondary metabolites known as macrocyclic trichothecenes that are harmful to human and animal health and affects at cellular level (Nielsen et al. 2001; Brasel et al. 2005; Kock et al, 2021). Several compounds in trichothecenes family have been isolated including trichodermin and trichodermol, which are toxic to several species of fungi (Ueno 1983 Hiratsuka et al. 1994; Hinkley et al. 2000; Skrobot et al. 2017).

The objectives of this study were to investigate (1) the population of fungal flora in outdoor environments in southern California and their occurrence in indoor environments, and (2) their colonisation and *in vitro* interactions in damp building materials.

## 2. Materials and methods

### 2.1. Sampling location and fungal flora

Outdoor and indoor samples were taken from 100 water-damaged houses in Los Angeles County between 1 June 2020 and 3 May 2021. According to the US National Weather Service, National Oceanic and Atmospheric Administration (www.noaa.gov), summer (June to August) temperature ranged from 35°C to 25°C during daytime and 18°C to 15°C during the evening. Autumn (September to November) temperature ranged from 30°C to 22°C during daytime and 18°C to 12°C during the evening. Winter (December to February) temperature ranged from 20°C to 18°C during daytime and 10°C to 8°C during the evening. Spring (March to May) temperature ranged from 25°C to 20°C during daytime and 15°C to 10°C during the evening. The average rainfall during study period was about 13 cm. Air samples for both outdoor and indoor environments was collected using Zefon air-o-cell sampler at a flow rate of 15 litres per minute (www.zefon.com). The air passes through a collection device (cassettes) which catches fungal spores embedded in a cover slip. This cover slip contains a sticky and optically clear sampling media which can permanently collect and hold fungal spores. The samples were collected twice a month for a period of one year. After being returned to the laboratory cassettes were opened, cover slips were removed and stained with cotton blue. The fungal spores were identified using Nikon Labophot-2 compound microscope with magnification up to 1000X based on their morphological characteristics (de Hoog et al. 2000; Samson et al. 2004; Domsch et al. 2007).

### 2.2. Fungal species and mycotoxin

The surface samples from 100 water-damaged houses were taken from drywall, ceiling tile and wood using a culture swab in the area where fungal growth can be seen. Five samples were taken from each building materials. Immediately after being returned to the laboratory the culture swabs were aseptically streaked out onto Petri plate in three different nutrient media (obtained from Hardy Diagnostics, 1430 W McCoy Lane, Santa Maria, CA 93455, USA). These media were malt extract agar with 0.1% chloramphenicol (Cat # W80), Dichloran-Glycerol-18 (Cat # W85), potato dextrose agar (Cat # W60) and carrot agar (carrot 250 g, yeast extract 1 g, agar 20 g, distilled water 1000 mL, pH 6.5). The plates were incubated at 25°C in the dark for 7 to 10 days. The fungal species from the Petri plates were surveyed through DNA metabarcoding analyses of the rDNA ITS2 region.

Six species of fungi were consistently isolated and identified through DNA metabarcoding analysis of the rDNA ITS2 region. These species were *Alternaria alternata, Aspergillus niger, Chaetomium globosum, Cladosporium herbarum, Penicillium chrysogenum* and *Stachybotrys chartarum*. Isolated and identified fungi were deposited at the fungal culture collection bank of the Pasteur laboratory.

Two trichothecenes, trichodermin (PLT07) and trichodermol (PLT09), were used in this study. Trichodermin and trichodermol were extracted from the culture *of S.chartarum. Stachybotrys chartarum* was grown in liquid malt extract (10-litre still culture) for 3 weeks. The mycelia were removed from the liquid culture by filtration through cheesecloth. The culture filtrate was passed through an XAD-16 column, which was then washed with acetone (1 litre). The acetone elute was concentrated under reduced pressure which was diluted with water and extracted with ethyl acetate three times. Removal of ethyl acetate under reduced pressure gave a yellow solid, which was then chromatographed on a Sephadex LH-20 column (acetone). The crude fraction was chromatographed on thin layer chromatography plates (1:1 acetone-hexane) and yielded two fractions. Chromatography of the first fraction on a silica gel column (3% acetone-dichloromethane) yielded 5.2 mg of trichodermin. Chromatography on the second fraction on a silica gel (5% methanol-dichloromethane) yielded 2.3 mg trichodermol. The spectroscopic data (IR, MS, ^1^H NMR, and ^13^C NMR) obtained for these two compounds were consistent with the literature (Ueno 1983).

### 2.3. In vitro *antagonism study*

Antagonism of *S. chartarum* against *A. alternata, A. niger, C. globosum, C. herbarum* and *P. chrysogenum* was studied on malt extract agar (MEA), potato dextrose agar (PDA) and carrot agar (CA) media in 90-mm Petri plates. *Stachybotrys chartarum* (a slower growing fungus) was inoculated with 5-mm agar plugs at the margin of the plate and allowed to grow at 25°C in the dark. For each medium, there were 15 replicates. Seven days later, 5-mm mycelial disks of *A. alternata, A. niger, C. globosum, C. herbarum* and *P. chrysogenum* from agar cultures were placed separately on the agar plates opposite to *S. chartarum* and the Petri plates were then incubated at described above. Colony diameter of the fungi was measured 6 days later. The inhibition zone formed around *A. alternata, A. niger, C. globosum, C. herbarum* and *P. chrysogenum* were measured after 6 days. Each of these inhibition zones were measured in a straight line from the edge of the *S. chartarum* colony to the edge of the *A. alternata, A. niger, C. globosum, C. herbarum* and *P. chrysogenum* colonies.

### 2.4. *Effect of culture filtrates of* S. chartarum *on the* in vitro *growth of five species of fungi in agar diffusion plates*

The effect of culture filtrates of *S. chartarum* on *A. alternata, A. niger, C. globosum, C. herbarum* and *P. chrysogenum* was studied on agar diffusion plates. These plates were prepared from 90-mm Petri plates containing 40 mL of MEA, PDA and CA media, by removing 5-mm-diameter agar plugs from each of four quarters of the plate. Five-mm-diameter agar plugs of *A. alternata, A. niger, C. globosum, C. herbarum* and *P. chrysogenum* were separately inoculated in the centre of the agar diffusion plates, and incubated at 25°C in the dark. The culture filtrate of *S. chartarum* was prepared by filtering 15-day-old liquid cultures (grown in carrot extract (CE) in 250 mL flasks on a shaker) through both Whatman No. 1 filter paper and a 0.45-μm Millipore filter and then drying on a rotary evaporator at 45°C. The evaporated sample was resuspended in 5-mL distilled water. One mL of filter sterilised concentrated culture filtrate of *S. chartarum* was added to diffusion wells of each of the 15 replicate plates containing 3-day-old cultures of *A. alternata, A. niger, C. globosum, C. herbarum* and *P. chrysogenum*. The plates were incubated at 25°C in the dark. After incubation for 7 days, the zone of inhibition formed around each diffusion well was measured. Microscopic examinations of hyphae of *A. alternata, A. niger, C. globosum, C. herbarum* and *P. chrysogenum* were also made.

### 2.5. *Effect of culture filtrates of* S. chartarum *on the* in vitro *mycelial growth of five species of fungi*

For this experiment, *S. chartarum* was grown in liquid malt extract (ME), potato dextrose broth (PDB) and CE at 25°C in the dark. After 15 days, the mycelia were harvested on Whatman No. 1 filter paper. The culture filtrate was collected and stored at 2°C in the dark overnight. Fifty mL of liquid media (ME, PDB, and CE) were autoclaved in 250-mL flasks for 15 min at 121°C. When cooled down, flasks were separately inoculated with five agar plugs (5 mm diameter) of actively growing mycelia of *A. alternata, A. niger, C. globosum, C. herbarum* and *P. chrysogenum*. The agar was removed with a sterile scalpel and only mycelial mats were inoculated in the flasks. Five mL of culture filtrate of *S. chartarum* was then added to each flask. The control consisted of 5 mL of sterile distilled water. There were 15 replicates for each treatment and each fungal species. The flasks were kept in the dark on a shaker at room temperature (24 ± 2°C). After 4-week incubation period, the mycelia of *A. alternata, A. niger, C. globosum, C. herbarum* and *P. chrysogenum* were harvested on a Whatman No. 1 filter paper, oven dried at 70°C for 48 h and the dry weight of mycelia calculated.

### 2.6. *Effect of trichodermin and trichdermol on the spore germination and* in vitro *growth of six species of fungi*

To test the effect of trichodermin and trichodermol on the spore germination, *A. alternata, A. niger, C. globosum, C. herbarum* and *P. chrysogenum* were grown on 2% MEA at 25°C in the dark for 1 week, whereas *S. chartarum* was grown on CA for 2 weeks. The spore suspension was prepared by transferring from fungal culture with a transfer loop into 9-mL sterile distilled water and the concentration of the suspension was adjusted to approximately 10^5^ spores/mL. Ten μL of spore suspension of *A. alternata, A. niger, C. globosum, C. herbarum, P. chrysogenum* and *S. chartarum* was mixed separately with 10 μL of filter sterilised trichodermin and trichodermol in a cavity slide. The slides with spores were kept moist by placing them on glass rods on the moistened filter paper in Petri plates and sealed with parafilm. There were 15 replicates for each treatment and each fungal species. Spore germination was recorded after 24 h incubation at 25°C in the dark, and 100 spores were counted for each treatment.

To test the effect of trichodermin and trichodermol on the *in vitro* growth of *A. alternata, A. niger, C. globosum, C. herbarum, P. chrysogenum* and *S. chartarum*, these fungi were grown on multiwell tissue culture plates (1.8 × 1.5 cm diameter × length of individual wells). Two percent MEA was used for *A. alternata, A. niger, C. globosum, C. herbarum* and *P. chrysogenum* and for *S. chartarum*, CA was used. Twenty-five μL of trichodermin and trichodermol at 1, 10, 100 and 1000 ppm in acetone-dichloromethane was added separately to the surface of the nutrient media in each well. For each concentration, 15 multiwells were used. In the control, only 25 μL of acetone-dichloromethane was used. All the multiwells were kept in a laminar flow hood for 1 min to allow the solvents to evaporate. Each agar well was individually inoculated with 5-mm agar plugs of *A. alternata, A. niger, C. globosum, C. herbarum, P. chrysogenum* and *S. chartarum* and then multiwells were wrapped with parafilm and incubated at 25°C in the dark. There were 15 replicates for each treatment and each fungal species. After 5 days of incubation, the colony diameters of *A. alternata, A. niger, C. globosum, C. herbarum*, and *P. chrysogenum* were measured and mycelia were observed under a microscope. For *S. chartarum* colony diameter and microscopic observation were made after 10 days.

### 2.7. *Effect of* S. chartarum *towards five fungal species on colonisation in the building materials*

#### 2.7.1. Growth of fungal species in the building materials

Three building materials (powdered drywall, powdered ceiling tile, and oak wood chips) were used in this study. The materials were crushed into coarse material using a grinder. One hundred gram of drywall, ceiling tile, and wood chips were soaked separately in ME in each of 95 flasks. After 1 h, the ME was drained and flasks were autoclaved for 60 min at 121°C. There were five replicates for each treatment and each fungal species. When cooled, 30 flasks containing dry wall, 30 flasks containing ceiling tile, and 30 flasks containing oak wood chips were separately inoculated with five agar plugs (5-mm diameter) of actively growing *A. alternata, A. niger, C. globosum, C. herbarum, P. chrysogenum* and *S. chartarum*. Five control flasks received five agar plugs (5-mm diameter) without any fungal species growing on it. The flasks were kept in the incubator at 25°C in the dark. After 45 days of incubation, flasks were removed from the incubator, observed under a microscope and isolation of the fungi was made from the inoculated drywall, ceiling tile and wood chips.

#### 2.7.2. *Interaction of* S. chartarum *on the growth of five fungal species in the building materials*

Three building materials described above were used in this study. One hundred gram of drywall, ceiling tile and wood chips were soaked separately in ME in each of eighty 250-mL flasks. After 1 h, the ME was drained and flasks were autoclaved for 60 min at 121°C. There were five replicates for each treatment. When cooled, 25 flasks containing dry wall, 25 flasks containing ceiling tile and 25 flasks containing oak wood chips were aseptically inoculated with five agar plugs (5-mm diameter) of actively growing mycelia of *S. chartarum*. The flasks were incubated at 25°C in the dark. The flasks were shaken periodically to fragment the mycelia of the fungi on the drywall, ceiling tile and wood chips. After 28 days, five flasks containing each of *S. chartarum* growing on drywall, ceiling tile and wood chips were separately inoculated with five agar plugs (5 mm diameter) of *A. alternata, A. niger, C. globosum, C. herbarum* and *P. chrysogenum* and returned to the incubator. Five control flasks contained only *S. chartarum* did not receive any treatment. The following treatments resulted: *S. chartarum* + *A. alternata, S. chartarum* + *A. niger, S. chartarum* + *C. globosum, S. chartarum* + *C. herbarum, S. chartarum* + *P. chrysogenum* and only *S. chartarum*. After 45 days of incubation, flasks were removed from the incubator, observed under a microscope, and isolation of the fungi was made from the inoculated drywall, ceiling tile and wood chips.

### 2.8. Statistical analysis

Data were subjected to analysis of variance (Zar 1984). Individual means were compared using Scheffe’s test for multiple comparisons using SAS software (SAS Institute Inc 2016). Means followed by the same letters (a,b,c and so on) in tables for a particular fungal species against *S. chartarum* and its metabolite trichodermin and trichodermol are not significantly (P = 0.05) different from each other by Scheffe’s test for multiple comparison.

## 3. Results

### 3.1. Fungal population

A total of 100 water-damaged houses were studied. From the outdoor air, a total of 42 fungal species and from indoor air 14 fungal species were consistently reported throughout the year (Table 1). There were no seasonal variations of these fungi throughout the year during study period.

**Table 1.** Occurrence of fungal flora in indoor and outdoor air and in damp building materials*.

Outdoor air	Indoor air	Damp building materials		
		Drywall	Wood	Ceiling tile
*Acremonium*	*Alternaria*	*Alternaria*	*Alternaria*	*Alternaria*
*Alternaria*	*Aureobasidium*	*Aspergillus*	*Aspergillus*	*Aspergillus*
*Arthrinium*	*Aspergillus*	*Chaetomium*	*Chaetomium*	*Chaetomium*
*Aspergillus*	*Chaetomium*	*Cladosporium*	*Cladosporium*	*Cladosporium*
*Aureobasidium*	*Cladosporium*	*Penicillium*	*Penicillium*	*Penicillium*
*Beltrania*	*Epicoccum*	*Stachybotrys*	*Stachybotrys*	*Stachybotrys*
*Bipolaris*	*Ganoderma*			
*Botrys*	*Memnoniella*			
*Chaetomium*.	*Mucor*			
*Cercospora*	*Nigrospora*			
*Cladosporium*.	*Periconia*			
*Curvularia*	*Phoma*			
*Emericella*	*Stachybotrys*			
*Epicoccum*	*Ulocladium*			
*Eurotium*				
*Fusarium*				
*Ganoderma*				
*Leptosphaeria*				
*Memnoniella*				
*Mucor*				
*Myrothecium*				
*Nigrospora*				
*Oidium*				
*Paecilomyces*				
*Penicillium*				
*Periconia*				
*Peronospora*				
*Peziza*				
*Phoma*				
*Pithomyces*				
*Polythrincium*				
*Phragmidium*				
*Rhizopus*				
*Spegazzinia*				
*Stachybotrys*				
*Stemphylium*				
*Taeniolella*				
*Tetraploa*				
*Torula*				
*Trichocladium*				
*Ulocladium*				
*Ustilago*				

*Samples were taken from inside and outside of 100 water-damaged houses in 2020–2021.

The fungal population and diversity were greater in outdoors than indoors. Indoor fungal species were reported from environments of water-damaged houses. Species of *Alternaria, Aspergillus, Chaetomium, Cladosporium, Penicillium* and *Stachybotrys* were always present in water-damaged building materials (Table 2). Species of *Alternaria* and *Cladosporium* were absent in the same substrate when *Stachybotrys* was present. On the other hand, species of *Aspergillus, Chaetomium* and *Penicillium* were associated *Stachybotrys* in these water-damaged building materials (Table 2). *Stachybotrys* was isolated from 83 water-damaged houses from the surface of the building materials, whereas species of *Alternaria, Aspergillus, Chaetomium, Cladosporium* and *Penicillium* were consistently isolated from all 100 houses in all three building materials.

**Table 2.** Coexistence of six most commonly fungal species in water-damaged building materials*.

Building materials	Occurrence of fungal species (when *Stachybotrys* was present in the same substrate)	Occurrence of fungal species (when *Stachybotrys* was absent in the same substrate)
Drywall	*Aspergillus,Chaetomium,Penicillium*, and *Stachybotrys.*	*Alternaria, Aspergillus, Chaetomium, Cladosporium*, and *Penicillium.*
Ceiling tile	*Aspergillus,Chaetomium,Penicillium*, and *Stachybotrys.*	*Alternaria, Aspergillus, Chaetomium, Cladosporium*, and *Penicillium.*
Wood	*Aspergillus,Chaetomium,Penicillium*, and *Stachybotrys.*	*Alternaria, Aspergillus, Chaetomium, Cladosporium*, and *Penicillium.*

*Samples were taken from 100 water damaged houses in 2020–2021. *Stachybotrys chartarum* was isolated from 83 water-damaged houses and other five fungal species were always present in all 100 water-damaged houses in 3 different building materials. Five samples were taken from each building material.

### 3.2. In vitro *antagonism study*

#### 3.2.1. *Inhibitory effect of* S. chartarum *towards five fungal species*

The *in vitro* growth of *A. alternata* and *C. herbarum* was significantly inhibited when grown in dual culture with *S. chartarum* on all three culture media used (Table 3). The inhibition zones, formed around the colonies of the fungi, ranged from 9.5 mm to 23.5 mm for *A. alternata* and 8.0 mm to 25.5 mm for *C. herbarum* in all three nutrient media tested (Table 3). *Aspergillus niger, C. globosum* and *P. chrysogenum* had no effect on their growth when grown together with or without *S. chartarum,* and no inhibition zones were formed around the colonies of *A. niger, C. globosum* and *P. chrysogenum* (Table 3).

**Table 3.** Inhibitory effect of *Stachybotrys chartarum* towards five fungal species on different nutrient media in agar plates.

Fungal species	Nutrient Media	Dual culture with *S. chartarum*		Without *S. chartarum*
		Colony diameter (mm)	Inhibition zone(mm))	Colony diameter (mm)
*A. alternata*	MEA	51.0^b^	9.5^c^	69.5^a^
	PDA	53.5^b^	17.0^c^	70.5^a^
	CA	50.5^b^	23.5^c^	60.0^a^
*A. niger*	MEA	90.0^a^	0.0^b^	90.0^a^
	PDA	89.0^a^	0.0^b^	88.0^a^
	CA	87.5^a^	0.0^b^	88.5^a^
*C. globosum*	MEA	40.5^a^	0.0^b^	42.0^a^
	PDA	36.0^b^	0.0^c^	41.5^a^
	CA	33.5^b^	0.0^c^	39.5^a^
*C. herbarum*	MEA	44.0^b^	8.0^c^	63.0^a^
	PDA	42.0^b^	21.0^c^	54.0^a^
	CA	36.5^b^	25.5^c^	46.5^a^
*P. chrysogenum*	MEA	89.5^a^	0.0^b^	90.0^a^
	PDA	90.0^a^	0.0^b^	89.0^a^
	CA	88.5^a^	0.0^b^	90.0^a^

Values are the means of 15 replicates. Means followed by the same letters (a,b,c) in a row for a particular species are not significantly (P = 0.05) different from each other by Scheffe’s test for multiple comparison. MEA, malt extract agar; PDA, potato dextrose agar; CA, carrot agar.

Similarly, in agar diffusion plates, inhibition zone was observed when *A. alternata* and *C. herbarum* were treated with culture filtrate of *S. chartarum* (Table 4). For *A. alternata* inhibition zone ranged from 6.5 mm to 25.5 mm and for *C. herbarum* it was 15.0 mm to 31.5 mm (Table 4). No inhibition zone was observed for *A. niger, C. globosum* and *P. chrysogenum* (Table 4). Control plates without culture filtrate of *S. chartarum* had no inhibition zones (Table 4).

**Table 4.** Inhibitory effect of culture filtrate of *Stachybotrys chartarum* towards five fungal species on different nutrient media in agar diffusion plates.

Fungal species	Nutrient Media	Culture Filtrate of *S. chartarum*	Control (Without Culture Filtrate of *S. chartarum*)
		Inhibition zone (mm)	Inhibition zone (mm)
*A. alternata*	MEA	6.5^b^	0.0^a^
	PDA	18.0^b^	0.0^a^
	CA	25.5^b^	0.0^a^
*A. niger*	MEA	0.0^a^	0.0^a^
	PDA	0.0^a^	0.0^a^
	CA	0.0 ^a^	0.0^a^
*C. globosum*	MEA	0.0^a^	0.0^a^
	PDA	0.0^a^	0.0^a^
	CA	0.0^a^	0.0^a^
*C. herbarum*	MEA	15.0^b^	0.0^a^
	PDA	28.0^b^	0.0^a^
	CA	31.5^b^	0.0^a^
*P. chrysogenum*	MEA	0.0^a^	0.0^a^
	PDA	0.0^a^	0.0^a^
	CA	0.0^a^	0.0^a^

Values are the means of 15 replicates. Means followed by the same letters (a,b) in a row for a particular species are not significantly (P = 0.05) different from each other by Scheffe’s test for multiple comparison. MEA, malt extract agar; PDA, potato dextrose agar; CA, carrot agar.

#### 3.2.2. *Effect of culture filtrates of* S. chartarum *on the* in vitro *mycelial growth of five species of fungi*

The mycelial growth of *A. alternata* and *C. herbarum* was significantly inhibited when treated with culture filtrate of *S. chartarum* (Table 5). Vacuolation of the hyphae of *A. alternata* and *C. herbarum* was observed under a microscope. The mycelial growth of *A. niger, C. globosum* and *P. chrysogenum* was not affected when grown with or without culture filtrate of *S. chartarum*. (Table 5). No vacuolation of the hyphae of *A. niger, C. globosum* and *P. chrysogenum* was observed under a microscope when grown together with culture filtrate of *S. chartarum*.

**Table 5.** Effect of culture filtrate of *Stachybotrys chartarum* on the *in vitro* mycelial growth (mg dry wt) of five fungal species on different nutrient media.

Fungal species	Without *S. chartarum* *(mg mycelial dry wt.)*			With *S. chartarum* *(mg mycelial dry wt.)*		
	ME	PDB	CE	ME	PDB	CE
*A. alternata*	5.0^b^	9.0^a^	6.5^b^	1.0^c^	1.5^c^	1.0^c^
*A. niger*	103.0^a^	96.0^b^	87.5^c^	104.0^a^	98.0^b^	86.5^c^
*C. globosum*	21.0^c^	30.5^a^	27.0^b^	22.5^c^	29.0^a^	25.5^b^
*C. herbarum*	19.5^a^	18.0^a^	19.0^a^	1.5^b^	1.0^b^	1.0^b^
*P. chrysogenum*	86.0^b^	91.0^a^	74.0^c^	85.0^b^	89.5^a^	73.0^c^

Values are the means of 15 replicates. Means followed by the same letters (a,b,c) in a row for a particular species are not significantly (P = 0.05) different from each other by Scheffe’s test for multiple comparison. ME, malt extract; PDB, potato dextrose broth; CE, carrot agar.

#### 3.2.3. *Effect of trichodermin and trichdermol on the spore germination and* in vitro *growth of five species of fungi*

At 1 ppm of trichodermin and trichodermol, spore germination of *A. alternata* and *C. herbarum* was not affected (Table 6). Spore germination of *A. alternata* and *C. herbarum* was significantly reduced when treated with trichodermin and trichodermol at 10, 100 and 1000 ppm (Table 6). Both these compounds had no effect on spore germination of *A. niger, C. globosum, P. chrysogenum* and *S. chartarum* at 1, 10, 100 and 1000 ppm (Table 6).

**Table 6.** Effect of trichodermin and trichodermol on the spore germination (%) of six fungal species.

Fungal species	Control	1 ppm	10 ppm	100 ppm	1000 ppm
Trichodermin:					
*A.alternata*	78^a^	76^a^	60^b^	6^c^	3^c^
*A. niger*	93^a^	94^a^	92^a^	90^a^	90^a^
*C. globosum*	84^a^	82^a^	81^a^	83^a^	81^a^
*C. herbarum*	81^a^	80^a^	39^b^	3^c^	1^c^
*P. chrysogenum*	91^a^	89^a^	90^a^	91^a^	90^a^
*S. chartarum*	63^a^	62^a^	61^a^	63^a^	62^a^
Trichodermol:					
*A.alternata*	71^a^	70^a^	28^b^	4^c^	1^c^
*A. niger*	90^a^	91^a^	92^a^	89^a^	90^a^
*C. globosum*	77^a^	75^a^	76^a^	77^a^	75^a^
*C. herbarum*	73^a^	71^a^	13^b^	1^c^	1^c^
*P. chrysogenum*	87^a^	85^a^	86^a^	85^a^	85^a^
*S. chartarum*	57^a^	57^a^	55^a^	56^a^	57^a^

Values are the means of 15 replicates. Means followed by the same letters (a,b,c) in a row for a particular species are not significantly (P = 0.05) different from each other by Scheffe’s test for multiple comparison

#### 3.2.4. *Effect of trichodermin and trichdermol on the* in vitro *mycelial growth of six species of fungi*

Both trichodermin and trichodermol at 1 ppm had no effect on the *in vitro* growth of *A. alternata* and *C. herbarum* (Table 7). *In vitro* growth of *A. alternata* and *C. herbarum* was significantly reduced when treated with trichodermin and trichodermol at 10, 100 and 1000 ppm (Table 7). Both these compounds had no effect on *in vitro* growth of *A. niger, C. globosum, P. chrysogenum* and *S. chartarum* at 1, 10, 100 and 1000 ppm (Table 7).

**Table 7.** Effect of trichodermin and trichodermol on the *in vitro* mycelial growth of six fungal species on different nutrient media.

% Reduction of growth from control									
Fungal species	Nutrient media	Trichodermin (ppm)				Trichodermol (ppm)			
		1	10	100	1000	1	10	100	1000
*A. alternata*	MEA	0.0^d^	13.0^b^	100^a^	100^a^	0.0^d^	5.5^c^	100^a^	100^a^
	PDA	0.0^d^	22.5^b^	100^a^	100^a^	0.0^d^	9.0^c^	100^a^	100^a^
	CA	0.0^d^	19.5^b^	100^a^	100^a^	0.0^d^	4.0^c^	100^a^	100 ^a^
*A. niger*	MEA	0.0^a^	0.0^a^	0.0^a^	0.0^a^	0.0^a^	0.0^a^	0.0^a^	0.0^a^
	PDA	0.0^a^	0.0^a^	0.0^a^	0.0^a^	0.0^a^	0.0^a^	0.0^a^	0.0^a^
	CA	0.0^a^	0.0^a^	0.0^a^	0.0^a^	0.0^a^	0.0^a^	0.0^a^	0.0^a^
*C. globosum*	MEA	0.0^a^	0.0^a^	0.0^a^	0.0^a^	0.0^a^	0.0^a^	0.0^a^	0.0^a^
	PDA	0.0^a^	0.0^a^	0.0^a^	0.0^a^	0.0^a^	0.0^a^	0.0^a^	0.0^a^
	CA	0.0^a^	0.0^a^	0.0^a^	0.0^a^	0.0^a^	0.0^a^	0.0^a^	0.0^a^
*C. herbarum*	MEA	0.0^d^	19.0^b^	100^a^	100^a^	0.0^d^	6.5^c^	100^a^	100^a^
	PDA	0.0^d^	10.5^b^	100^a^	100^a^	0.0^d^	3.0^c^	100^a^	100^a^
	CA	0.0^d^	13.5^b^	100^a^	100 ^a^	0.0^d^	2.0^c^	100^a^	100^a^
*P. chrysogenum*	MEA	0.0^a^	0.0^a^	0.0^a^	0.0^a^	0.0^a^	0.0^a^	0.0^a^	0.0^a^
	PDA	0.0^a^	0.0 ^a^	0.0^a^	0.0^a^	0.0^a^	0.0^a^	0.0^a^	0.0^a^
	CA	0.0^a^	0.0 ^a^	0.0^a^	0.0^a^	0.0^a^	0.0^a^	0.0^a^	0.0^a^
*S. chartarum*	MEA	0.0^a^	0.0^a^	0.0^a^	0.0^a^	0.0^a^	0.0^a^	0.0^a^	0.0^a^
	PDA	0.0^a^	0.0^a^	0.0^a^	0.0^a^	0.0^a^	0.0^a^	0.0^a^	0.0^a^
	CA	0.0^a^	0.0^a^	0.0^a^	0.0^a^	0.0^a^	0.0^a^	0.0^a^	0.0^a^

Values are the means of 15 replicates. Means followed by the same letters (a,b,c and so on) for trichodermin and trichodermol for a particular fungal species in a row are not significantly (P = 0.05) different from each other by Scheffe’s test for multiple comparison. MEA, malt extract agar; PDA, potato dextrose agar; CA, carrot agar.

#### 3.2.5. *Effect* of S. chartarum *on five fungal species on colonisation in building materials*

All the six species of fungi grew well in drywall, ceiling tile and oak wood chips (Table 8). Both *A. alternata* and *C. herbarum* failed to grow in these substrate when grown together with *S. chartarum* (Table 8). The growth of A. *niger*, *C. globosum* and *P. chrysogenum* was not inhibited when grown together with *S. chartarum* in these substrate (Table 8).

**Table 8.** Growth of six fungal species and interaction of *Stachybotrys chartarum* with other fungal species in the building materials.

Fungal species	Dry wall	Ceiling tile	Oak wood chips
*A. alternata*	+++	+++	+++
*A. niger*	+++	+++	+++
*C. globosum*	+++	+++	+++
*C.herbarum*	+++	+++	+++
*P. chrysogenum*	+++	+++	+++
*S. chartarum*	+++	+++	+++
*A. alternata* + *S. chartarum*	+	+	+
*A. niger* + *S. chartarum*	++	++	++
*C. globosum* + *S. chartarum*	++	++	++
*C.herbarum* + *S. chartarum*	+	+	+
*P. chrysogenum* + *S. chartarum*	++	++	++

+++ = Growth of fungi; ++ = Growth of both *S. chartarum* and other fungi in combination; + = Growth of *S. chartarum* only in combination.

## 4. Discussion

The present study shows that a higher number of fungal species present in the outdoor environments, and there is a significant difference between the fungal populations in both indoor and outdoor environments. Indoor fungal flora is thought to be a function of dispersal from the outdoor environment and growth and resuspension from the indoor environment (Ara et al. 2004; Horner et al. 2004; Adams et al. 2013; Atya et al. 2019; Andersen et al. 2021).

The source of fungi that grow indoors is from the outside environment, and many of these fungi are capable of finding suitable growth conditions in indoor environments. During this study, species of *Aspergillus* and *Stachybotrys* are found in both outdoor and indoor environments; however, Hegarty et al. (2019) found these two species occur only in the indoor environment. Present study shows indoor environment supported the growth of water-indicator fungi as well as leaf and soil-borne fungi. Estensmo et al. (2021) concluded from their study that indoor fungal flora are structured by occupancy as well as outdoor seasonality and temporal variability should be accounted for indoor air quality study. Modern building materials, once moistened, may provide rich ecological niches for various fungi. It has long been postulated that damp or homes with high humidity have a musty smell or have obvious fungal growth. Building materials in southern California consist wood, drywall, insulation, ceiling tile and these materials provide excellent food source for fungi. Once spores of fungi land on these damp building materials, spores will germinate, grow and multiply within a few weeks. The occupants of these homes have increase in subjective complaints including upper respiratory, asthma, gastrointestinal and other illness (Davies et al. 1995; Anderson et al. 1997; Taskinen et al. 1997., 1999).

In this study, we have found that species of *Alternaria, Aspergillus, Chaetomium, Cladosporium, Penicillium* and *Stachybotrys* grow luxuriantly in these substrata. These fungi are water indicator and present when there is food source and high humidity. It was interesting to note that *A. alternata* and *C. herbarum* grow in the same substrate when *S. chartarum* was absent and failed to grow when *S. chartarum* was present. On the other hand, colonies of *A. niger, C. globosum* and *P. chrysogenum* were often associated with *S. chartarum*.

*In vitro* inhibition of fungi has been attributed through parasitism or surface contact followed by penetration and formation of intercellular hyphae within the host hyphae, or production of antifungal compounds by antagonist fungus (Demain 1984). In this study, *S. chartarum* was an effective antagonist against *A. alternata* and *C. herbarum* owing to the production of antifungal substances that were excreted in the culture media. *Stachybotrys chartarum* was capable of inhibiting growth of both *A. alternata* and *C. herbarum*. Although *S. chartarum* did not parasitise or penetrate the hyphae of *A. alternata* and *C. herbarum*, the metabolites produced by *S. chartarum* appeared to kill these two fungi. The cytoplasm of *A. alternata* and *C. herbarum* coagulated and disintegrated when exposed to the *S. chartarum* culture, its culture filtrate, and trichodermin and trichodermol. In this study, mycelial growth of *A. alternata* and *C. herbarum* was also inhibited in both agar and liquid culture by *S. chartarum*. The crude extract of *S. chartarum* also strongly reduced the growth of *A. alternata* and *C. herbarum*. These results indicate that inhibition of *A. alternata* and *C. herbarum* by *S. chartarum* was not hyphal parasitism rather it is a chemical in nature.

All these six fungal species described above are known to produce mycotoxin. Mycotoxins are the secondary metabolites of fungi that represent a chemically diverge group of organic, non-volatile, and low molecular weight compounds. Mycotoxins are usually produced when conditions favour fungal growth such as moisture, pH, growth medium, and temperature. Among these fungi, *S. chartarum* is considered to be one of the most toxic fungi and produce cytotoxic compound known as trichothecene. Trichothecenes are secondary metabolites produced by species of *Stachybotrys* as well as other fungi that are harmful to human and animal health causing wide range of diseases (D’Mello et al. 1999; Shifrin and Anderson 1999; Gutleb 2002; Skrobot et al. 2017). There are over 150 trichothecenes and trichothecenes derivatives have been isolated and characterised (Gutleb et al. 2002). They are all non-volatile, low molecular weight sesquiterpene epoxides and share a tricyclic nucleus and usually contain an epoxide at C-12 and C-13, which are essential for toxicity (Desjardins et al. 1993). The trichothecene skeleton is chemically stable and not degraded by heat or neutral or acidic pH (Eriksen 2003). In this study, two trichothecene, trichodermin and trichodermol produced by species of *Stachybotrys* showed significantly inhibitory effect on *A. alternata* and *C. herbarum* at 10, 100 and 1000 ppm. Both spore germination and mycelial growth of these fungi were significantly inhibited by these compounds. Interestingly, *A. niger, C. globosum, P. chrysogenum* and *S. chartarum* were not affected by any concentration of trichodermin and trichodermol up to 1000 ppm. This indicates that the effect of these trichothecenes varied considerably amongst different fungal species.

The antifungal activity of *S. chartarum* was also confirmed on drywall, ceiling tile and oak wood chips where it prevented the growth of *A. alternata* and *C. herbarum* in these substrates. For antibiotic metabolite production to occur in these substrate, certain conditions for secondary metabolism would have to met. Secondary metabolite formation usually occurs after a period of hyphal growth has taken place, thus establishing a certain hyphal mass, age, or growth rate. It is interesting to note that growth of *S. chartarum* was not affected by its own metabolite. Both *A. alternata* and *C. herbarum* colonised abundantly in building materials in the same houses when *S. chartarum* was absent, however, they failed to colonised in the same building materials when *S. chartarum* was present.

From this study, distinct interaction patterns between *S. chartarum* against *A. alternata* and *C. herbarum* was identified and determined by the antifungal compounds production by *S. chartarum*. Trichodermin and trichodermol were toxic to both *A. alternata* and *C. herbarum* at concentrations 10 ppm and above but had no effect on *A. niger, C. globosum* and *P. chrysogenum* up to 1000 ppm. This may explain why *A. alternata* and *C. herbarum* were absent in the same building materials when colonised by *S. chartarum* but the growth *A. niger, C. globosum* and *P. chrysogenum* was not affected.

This study shows that there exist an associated mycobiota on the damp building materials based on interactions between these fungi. The first association of fungi was between *A. alternata, A. niger, C. globosum, C. herbarum* and *P. chrysogenum*. Although all the fungi produce mycotoxins, these fungi have found their niche on damp or wet building materials without inhibiting growth of each other. A second strong association was seen between mycotoxin producing fungi *A. niger, C. globosum, P. chrysogenum* and *S. chartarum,* and they seem to coexist together on damp or wet building materials.

## 5. Conclusions

Fungal population varied significantly in outdoor and indoor damp environments and certain fungi occur in both the environments. In damp houses, fungal population can reach or exceed those of outdoor levels causing indoor fungal contamination. Modern building materials, once moistened, may provide rich ecological niches for various fungi. It has long been postulated that damp or homes with high humidity have a musty smell or have obvious fungal growth. Indoor fungal flora is thought to be a function of dispersal from the outdoor environment and growth and resuspension from the indoor environment. The source of fungi that grow indoors is from the outside environment and many of these fungi are capable of finding suitable growth conditions in indoor environments. In this study, indoor environment supported the growth of water-indicator fungi as well as leaf and soil-borne fungi. Six commonly occurring fungal species in damp or water-damaged houses in southern California are *A. alternata, A. niger, C. globosum, C. herbarum* and *P. chrysogenum* and *S. chartarum. Stachybotrys chartarum* was a strong antagonist against *A. alternata* and *C. herbarum* but had no inhibitory effect on the growth of *A. niger, C. globosum*, and *P. chrysogenum*. Two trichothecenes, produced by *S. chartarum*, trichodermin and trichodermol, significantly inhibited spore germination and *in vitro* growth of *A. alternata* and *C. herbarum* but had no inhibitory effect on *A. niger, C. globosum, P. chrysogenum* and *S. chartarum. Stachybotrys chartarum* significantly inhibited the growth of *A. alternata* and *C. herbarum* in the building materials (drywall, ceiling tile and oak woods) and had no effect on the growth and colonisation of *A. niger, C. globosum* and *P. chrysogenum*. There seemed to appear two associations of fungi in the damp or water damaged building materials. The first association of fungi was between *A. alternata, A. niger, C. globosum, C. herbarum* and *P. chrysogenum*. A second strong association was seen between *A. niger, C. globosum, P. chrysogenum* and *S. chartarum* and they seem to coexist together.
